# Family Ties at End-of-Life: Characteristics of Nursing Home Decedents With and Without Family

**DOI:** 10.1089/pmr.2023.0023

**Published:** 2023-11-16

**Authors:** Caroline E. Stephens, Djin Tay, Eli Iacob, Michael Hollinghaus, Rebecca Goodwin, Brenna Kelly, Ken Smith, Lee Ellington, Rebecca Utz, Katherine Ornstein

**Affiliations:** ^1^ College of Nursing, University of Utah, Salt Lake City, Utah, USA.; ^2^ Kem C. Gardner Policy Institute, University of Utah, Salt Lake City, Utah, USA.; ^3^ School of Medicine, Department of Population Health Sciences, University of Utah, Salt Lake City, Utah, USA.; ^4^ Huntsman Cancer Institute, University of Utah, Salt Lake City, Utah, USA.; ^5^ College of Social & Behavioral Sciences, Department of Family and Consumer Studies, University of Utah, Salt Lake City, Utah, USA.; ^6^ College of Social & Behavioral Sciences, Department of Sociology, University of Utah, Salt Lake City, Utah, USA.; ^7^ Johns Hopkins School of Nursing, Center for Equity in Aging, Baltimore, Maryland, USA.

**Keywords:** death, end of life, family, hospitalization, nursing home, rural

## Abstract

**Background:**

Little is known about nursing home (NH) residents' family characteristics despite the important role families play at end-of-life (EOL).

**Objective:**

To describe the size and composition of first-degree families (FDFs) of Utah NH residents who died 1998–2016 (*n* = 43,405).

**Methods:**

Using the Utah Population Caregiving Database, we linked NH decedents to their FDF (*n* = 124,419; spouses = 10.8%; children = 55.3%; siblings = 32.3%) and compared sociodemographic and death characteristics of those with and without FDF members (*n* = 9424).

**Results:**

Compared to NH decedents with FDF (78.3%), those without (21.7%) were more likely to be female (64.7% vs. 57.1%), non-White/Hispanic (11.2% vs. 4.2%), less educated (<9th grade; 41.1% vs. 32.4%), and die in a rural/frontier NH (25.3% vs. 24.0%, all *p* < 0.001). Despite similar levels of disease burden (Charlson Comorbidity score 3 + 37.7% vs. 38.0%), those without FDF were more likely to die from cancer (14.2% vs. 12.4%), Chronic Obstructive Pulmonary Disease (COPD) (6.0% vs. 4.0%), and dementia (17.1% vs. 16.6%, all *p* < 0.001), and were less likely to have 2+ hospitalizations at EOL (20.5% vs. 22.4%, *p* < 0.001).

**Conclusions:**

Among NH decedents, those with and without FDF have different sociodemographic and death characteristics—factors that may impact care at EOL. Understanding the nature of FDF relationship type on NH resident EOL care trajectories and outcomes is an important next step in clarifying the role of families of persons living and dying in NHs.

## Introduction

Nursing homes (NHs) serve as the primary site of end-of-life (EOL) care for persons with chronic progressive illnesses.^1,2^ Studies find that one in five Americans, including nearly 70% of people with advanced dementia, die in NHs each year.^2,3^ By 2030, 40% of all U.S. deaths are projected to occur in NHs.^3,4^ Unfortunately, many NH residents have unmet needs for symptom management and emotional support, poor resident/family-provider communication, and experience burdensome care transitions at EOL.^5–8^

Families often play a critical role in person-centered care for NH residents, promoting quality of life and making EOL care decisions.^9,10^ They may visit more frequently and provide more hands-on support when concerned about the resident's well-being or when they lack confidence in the care provided.^9,10^ Additionally, families may commonly react to changes in resident condition as a crisis, leading them to advocate for care that may be physically and psychologically detrimental,^5,6^ particularly during the final months of life. Many NH residents, however, do not have any family to advocate for their care, which may impact their EOL care.^11,12^ Despite the influential role of families at EOL, little is known about the characteristics of families of those who die in a NH. The purpose of this brief report is to describe the sociodemographic and death characteristics of NH decedents with and without first-degree family (FDF) members.

## Methods

This retrospective cohort study focused on 43,405 individuals in the Utah Caregiving Population Science (Utah C-PopS) cohort^13,14^ who died in a Utah NH of natural causes between 1998 and 2016. The Utah C-PopS cohort is derived from the Utah Population Database^15–18^ which is a statewide population health research database that links individual-level administrative and health data to spousal and FDF members' data through a population pedigree (genealogy) structure. FDF include children, siblings, and parents. Key variables and data sources used to measure decedent sociodemographic, EOL care, and death characteristics are described elsewhere.^13^ Descriptive statistics, chi-square tests, and *t*-tests were performed to describe the NH decedent cohort and their FDF members, as well as to compare sociodemographic, socioeconomic, and death characteristics of NH decedents with and without FDF. All descriptive statistics analyses were performed using R statistical software (Core Team, 2021). This study was approved by the University of Utah's Resource for Genetic and Epidemiologic Research and Institutional Review Board.

## Results

### Characteristics of NH decedents

**FIG. 1.**
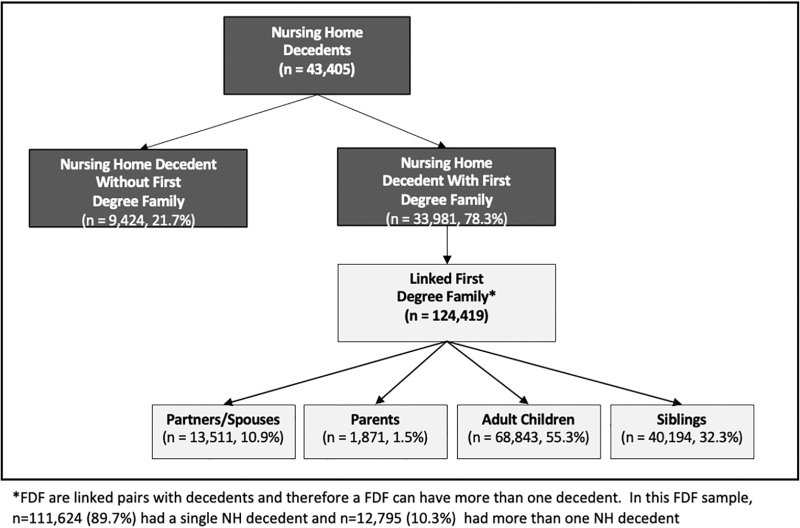
NH decedent cohort with and without FDF members (*n* = 43,405) and linkages of the UPDB data sources. FDF, first-degree family; NH, nursing home; UPDB, Utah Population Database.

Of the 43,405 individuals who died in a Utah NH of natural causes between 1998 and 2016, 22% (*n* = 9424) did not have any FDF members and 78% (*n* = 33,981) were linked to ≥1 adult FDF (*n* = 124,419; spouses = 10.9%; parents = 1.5%; children = 55.3%; siblings = 32.3%; Fig. 1). The median age at death of NH residents was 84.7 years (interquartile range [IQR] 20.80–99.99). The majority were female (58.7%), White/non-Hispanic (94.3%), widowed (51.1%), with a high school education (36.5%), living in an urban NH at the time of their death (75.7%) (Table 1).

**Table 1.** Demographic, Socioeconomic, Context of Death, End-of-Life Health Care Utilization, and Size and Characteristics of First-Degree Family of Nursing Home Decedents

	Female^a^	Male	Total
	(***n*** = 25,495) ***n*** (%)	(***n*** = 17,940) ***n*** (%)	(***n*** = 43,405) ***n*** (%)
Demographic characteristics			
Age at decedents' death^b^			
Mean (±SD)^**^	83.79 (10.48)	80.37 (11.44)	82.38 (11.02)
Median (range)	85.92 (20.80–99.9)	82.90 (21.68–99.98)	84.70 (20.80–99.99)
Race/Ethnicity^**^^,c^			
Hispanic	775 (3.0)	684 (3.8)	1459 (3.4)
Non-White non-Hispanic	534 (2.1)	501 (2.8)	1035 (2.4)
White non-Hispanic	24,186 (94.9)	16,725 (93.4)	40,911 (94.3)
Marital status^**^^,d^			
Divorced/separated	3288 (12.9)	2663 (14.9)	5951 (13.7)
Married	4145 (16.3)	8639 (48.2)	12,784 (29.5)
Others	1225 (4.8)	1245 (7.0)	2470 (5.7)
Widowed	16,837 (66.0)	5363 (29.9)	22,200 (51.1)
Socioeconomic characteristics			
Maximum education^**^^,e^			
College graduate	1257 (4.9)	1271 (7.1)	2528 (5.8)
High school graduate	9936 (39.0)	5895 (32.9)	15,831 (36.5)
Less than high school	9388 (36.8)	5489 (30.6)	14,877 (34.3)
Post college	500 (2.0)	1210 (6.8)	1710 (3.9)
Some college	4414 (17.3)	4045 (22.6)	8459 (19.5)
Region^f^			
Frontier	1046 (4.1)	742 (4.1)	1788 (4.1)
Rural	5061 (19.9)	3718 (20.8)	8779 (20.2)
Urban	19,388 (76.0)	13,450 (75.1)	32,838 (75.7)
Insurance status^**^^,g^			
Medicaid only	582 (2.3)	567 (3.2)	1149 (2.6)
Medicare and Medicaid	4006 (15.7)	2145 (12.0)	6151 (14.2)
Medicare only	12,525 (49.1)	9942 (55.5)	22,467 (51.8)
No record of either	8382 (32.9)	5256 (29.3)	13,638 (31.4)
Context of death and EOL health care utilization			
Primary cause of death^**^^,h^			
Cancer	2611 (10.2)	2932 (16.4)	5543 (12.8)
Cerebrovascular disease	2349 (9.2)	1360 (7.6)	3709 (8.5)
COPD	1030 (4.0)	1025 (5.7)	2055 (4.7)
Dementia	4777 (18.7)	2477 (13.8)	7254 (16.7)
Heart disease	5551 (21.8)	3591 (20.1)	9142 (21.1)
Others	9177 (36.0)	6525 (36.4)	15,702 (36.2)
Diagnosis of Dementia^**^	11,541 (45.3)	7259 (40.5)	18,800 (43.3)
Comorbidities-CCI^**^^,i^			
0	3239 (12.7)	1644 (9.2)	4883 (11.2)
1–2	9039 (35.5)	5788 (32.3)	14,827 (34.2)
3–4	5003 (19.6)	4397 (24.6)	9400 (21.7)
5+	3218 (12.6)	3842 (21.5)	7060 (16.3)
Unknown^j^	4996 (19.6)	2239 (12.5)	7235 (16.7)
Inpatient Hospitalization at EOL^**^			
One within six months	7403 (29.0)	5877 (32.8)	13,280 (30.6)
Two or more within six months	4875 (19.1)	4667 (26.1)	9542 (22.0)
None within six months	4965 (19.5)	3270 (18.3)	8235 (19.0)
None within two years	8252 (32.4)	4096 (22.9)	12,348 (28.4)
Any inpatient hospitalization within last month^**^	5912 (23.2)	5475 (30.6)	11,387 (26.2)
Size of FDF (parent, child, sibling, spouses)^**^			
0	6096 (23.9)	3328 (18.6)	9424 (21.7)
1	4107 (16.1)	3070 (17.1)	7177 (16.5)
2	3634 (14.3)	2258 (12.6)	5892 (13.6)
3	3285 (12.9)	2239 (12.5)	5524 (12.7)
4–5	4774 (18.7)	3634 (20.3)	8408 (19.4)
6–10	3396 (13.3)	3170 (17.7)	6566 (15.1)
11+	203 (0.8)	211 (1.2)	414 (1.0)
Average number of FDFs (SD)^**^	2.70 (2.51)	3.10 (2.70)	2.87 (2.60)
Median number of FDF (Q1–Q3)^**^	2 (0–18)	3 (0–26)	2 (0–26)
Type and number of FDF members			
Spouse, *n* (%)^**^	4861 (19.1)	8645 (48.3)	13,506 (31.1)
Parent, *n* (%)^**^	676 (2.7)	809 (4.5)	1485 (3.4)
Child, *n* (%)^**^	15,635 (61.3)	10,583 (59.1)	26,218 (60.4)
Sibling, *n* (%)^**^	10,987 (43.1)	8074 (45.1)	19,061 (43.9)
Children, mean (SD)	1.58 (1.72)	1.59 (1.80)	1.59 (1.76)
Children, median (range)	1 (0–12)	1 (0–25)	1 (0–25)
Siblings, mean (SD)^**^	0.89 (1.36)	0.97 (1.43)	0.93 (1.39)
Siblings, median (range)^**^	0 (0–11)	0 (0–13)	0 (0–13)

^a^ *Sex* as reported from UPDB. Unknown sex was excluded due to small numbers with the decedent and was obtained from the death certificate.

^b^ *Age* of decedent was referenced at the approximate time of the decedents' death.

^c^ *Race and Ethnicity* were based on the National Institute of Health definitions. Unknown race/ethnicity was excluded due to small numbers.

^d^ *Marital status* was defined as having a legal relationship with the decedent and was obtained from the death certificate. Unknown marital status was excluded due to small numbers.

^e^ *Education*—maximum level of education for decedents as reported from the UPDB was obtained. Unknown education was excluded due to small numbers.

^f^ *Region*—region was derived from zip code of decedents' residence as reported in death certificates and was categorized using 2018 Utah Department of Health classifications. Urban counties have a population density of >100 people per sq. mile; rural counties have a population density of <99 and >6 people per sq. mile, and Frontier counties have <6 people per sq. mile.

^g^ *Insurance coverage* was only available for individuals who had an inpatient or ambulatory surgery or emergency record in the UPDB within the last year of decedents' death. Please note that inpatient and ambulatory surgery records were from 1996 to 2016 and emergency department records were from 2000 to 2016. Medicare only means evidence of Medicare but no Medicaid; Medicaid only evidence of Medicaid but no Medicare.

^h^ *Primary cause of death* was obtained from death records and defined with ICD-9 and ICD-10 codes.

^i^ *Comorbidity*—Consistent with Charlson et al.,^20^ we used a weighted CCI that takes into account the number and seriousness of comorbid disease. Each comorbidity was weighted by a value, depending on the prognostic impact of a comorbid condition, ranging from 1 to 6 points with scores summed to calculate a CCI. Based on the CCI score, the severity of comorbidity was categorized into three grades: mild, with CCI scores of 1–2; moderate, with CCI scores of 3–4; and severe, with CCI scores ≥5. The CCI was based on diagnosis data obtained from inpatient, ambulatory surgery, and emergency department records within the last two years of decedents' death (Note: inpatient and ambulatory surgery records were from 1996 to 2016; emergency department records were from 2000 to 2016).

^j^ *Decedents* did not have any inpatient/ambulatory surgery/emergency records in UPDB within two years before death.

*p*-Values were calculated from chi-square tests for categorical variables and *t*-tests for continuous variables. ^*^p < 0.05, ^**^p < 0.01.

CCI, Charlson Comorbidity Index; COPD, Chronic Obstructive Pulmonary Disease; EOL, end-of-life; FDF, first-degree family; ICD, International Classification of Diseases; SD, standard deviation; UPDB, Utah Population Database.

Nearly 40% had moderate levels of comorbidity (Charlson Comorbidity Index [CCI] 3+),^19,20^ 43.3% had a diagnosis of dementia, and almost a quarter had two or more hospitalizations in their last six months of life. Males had more inpatient hospitalizations within the last month of life compared to females (30.6% vs. 23.2%, *p* < 0.0001). The most common primary causes of death were heart disease (21.1%), dementia (16.7%), and cancer (12.8%).

The median number of FDF members for NH decedents was two (IQR = 0–26). Male decedents were more likely to have at least one documented FDF compared to females (81.4% vs. 76.1%, *p* < 0.001) and a spouse (48.3% vs. 19.1%, *p* < 0.001).

### NH decedents' FDF characteristics

Figure 1 and Table 2 describe the characteristics of NH decedents' FDF members by relationship type (*n* = 124,419). The median age of FDF members at time of decedent death was 63.4 (IQR 20.01–108.85). The majority of FDF members were female (52.6%), White/non-Hispanic (95.0%), married (60.9%), with high school/some college education (53.6%), and lived in an urban setting (75.2%).

**Table 2.** Demographic and Socioeconomic Characteristics of Nursing Home Decedents for First Degree Family Members

	Children	Parents	Siblings	Spouses	Total FDF
	(***n*** = 68,843) ***n*** (%)	(***n*** = 1871) ***n*** (%)	(***n*** = 40,194) ***n*** (%)	(***n*** = 13,511) ***n*** (%)	(***n*** = 124,419) ***n*** (%)
Demographic characteristics					
Age at decedents' death					
Mean (±SD)^**^	54.61 (10.24)	80.56 (10.87)	75.94 (11.82)	78.89 (10.11)	64.53 (15.45)
Median (range)^**^	55.46 (20.01, 82.18)	80.56 (38.05, 108.85)	77.90 (20.29, 104.94)	80.65 (21.50, 101.92)	63.42 (20.01, 108.85)
Gender^**^					
Female	33,515 (48.7)	1233 (65.9)	22,029 (54.8)	8649 (64.0)	65,426 (52.6)
Male	35,328 (51.3)	638 (34.1)	18,165 (45.2)	4862 (36.0)	58,993 (47.4)
Race/Ethnicity^**^^,a^					
Hispanic	2631 (3.8)	89 (4.8)	839 (2.1)	368 (2.7)	3927 (3.2)
Non-White non-Hispanic	1485 (2.2)	38 (2.0)	468 (1.2)	284 (2.1)	2275 (1.8)
White non-Hispanic	64,727 (94.0)	1744 (93.2)	38,887 (96.7)	12,859 (95.2)	118,217 (95.0)
Marital status^**^					
Divorced/separated	8057 (11.7)	130 (6.9)	2494 (6.2)	86 (0.6)	10,767 (8.7)
Married	48,692 (70.7)	786 (42.0)	22,187 (55.2)	4094 (30.3)	75,759 (60.9)
Others	9444 (13.7)	68 (3.6)	3017 (7.5)	166 (1.2)	12,695 (10.2)
Widowed	2650 (3.8)	887 (47.4)	12,496 (31.1)	9165 (67.8)	25,198 (20.3)
Socioeconomic characteristics					
Maximum education^**^					
Less than high school	4683 (6.8)	608 (32.5)	10,353 (25.8)	3447 (25.5)	19,091 (15.3)
High school graduate	19,615 (28.5)	604 (32.3)	13,278 (33.0)	4345 (32.2)	37,842 (30.4)
Some college	18,425 (26.8)	295 (15.8)	7603 (18.9)	2532 (18.7)	28,855 (23.2)
College graduate	8207 (11.9)	102 (5.5)	2631 (6.5)	864 (6.4)	11,804 (9.5)
Post college	7947 (11.5)	55 (2.9)	2012 (5.0)	468 (3.5)	10,482 (8.4)
Unknown	9966 (14.5)	207 (11.1)	4317 (10.7)	1855 (13.7)	16,345 (13.1)
Region^**^					
Frontier	2923 (4.2)	91 (4.9)	2254 (5.6)	551 (4.1)	5819 (4.7)
Rural	11,972 (17.4)	310 (16.6)	8147 (20.3)	2902 (21.5)	23,331 (18.8)
Unknown	1166 (1.7)	17 (0.9)	486 (1.2)	99 (0.7)	1768 (1.4)
Urban	52,782 (76.7)	1453 (77.7)	29,307 (72.9)	9959 (73.7)	93,501 (75.2)

^**^ *p* < 0.01.

^a^ *Race/ethnicity* was utilized rather than race categories due to privacy concerns with cell sizes <10.

### Comparison of characteristics of decedents with and without FDF

Compared to NH decedents with FDF members, those without were more likely to be older (median age 85.3 vs. 84.6), female (64.7% vs. 57.1%), non-White/Hispanic (11.2% vs. 4.2%), less educated (<9th grade; 41.1% vs. 32.4%), have Medicaid only (4.0% vs. 2.3%), and die in a rural/frontier NH (25.3% vs. 24.0%, all *p* < 0.001, Table 3). NH decedents without FDF were also more likely to die from cancer (14.2% vs. 12.4%), Chronic Obstructive Pulmonary Disease (COPD) (6.0% vs. 4.0%), and dementia (17.1% vs. 16.6%, all *p* < 0.001), compared to NH decedents with FDF. While NH residents with and without FDF members had similar levels of disease burden (CCI score 3 + 37.7% vs. 38.0%), NH residents without any FDF members were less likely to have two or more hospitalizations at EOL (20.5% vs. 22.4%, *p* < 0.001).

**Table 3.** Bivariate Analysis of Demographic, Socioeconomics, Context of Death, and End-of-Life Health Care Utilization Differences Between Nursing Home Decedents With and Without First-Degree Family

	No FDF	Any FDF
	(***n*** = 9424) ***n*** (%)	(***n*** = 33,981) ***n*** (%)
Characteristics of decedents		
Age at decedents' death		
Mean (±SD)^**^	82.58 (11.56)	82.33 (10.86)
Median (range)^**^	85.26 (23.77, 99.93)	84.56 (20.80, 99.99)
Gender^**^		
Female	6096 (64.7)	19,399 (57.1)
Male	3328 (35.3)	14,582 (42.9)
Race/Ethnicity^**^^,a^		
Hispanic	576 (6.1)	883 (2.6)
Non-White non-Hispanic	476 (5.1)	559 (1.6)
White non-Hispanic	8372 (88.8)	32,539 (95.8)
Marital status^**^		
Divorced/separated	2054 (21.8)	3897 (11.5)
Married	747 (7.9)	12,037 (35.4)
Others	1084 (11.5)	1386 (4.1)
Widowed	5539 (58.8)	16,661 (49.0)
Socioeconomic characteristics		
Maximum education^**^		
Less than high school	3871 (41.1)	11,006 (32.4)
High school graduate	3045 (32.3)	12,786 (37.6)
Some college	1675 (17.8)	6784 (20.0)
College graduate	508 (5.4)	2020 (5.9)
Post college	325 (3.4)	1385 (4.1)
Region^**^		
Frontier	345 (3.7)	1443 (4.2)
Rural	2035 (21.6)	6744 (19.8)
Urban	7044 (74.7)	25,794 (75.9)
Insurance status^**^		
Medicaid only	373 (4.0)	776 (2.3)
Medicare and Medicaid	1918 (20.4)	4233 (12.5)
Medicare only	4065 (43.1)	18,402 (54.2)
No record of either	3068 (32.6)	10,570 (31.1)
Context of death and EOL health care utilization		
Primary cause of death^**^		
Cancer	1338 (14.2)	4205 (12.4)
Cerebrovascular disease	737 (7.8)	2972 (8.7)
COPD	568 (6.0)	1487 (4.4)
Dementia	1615 (17.1)	5639 (16.6)
Heart disease	1955 (20.7)	7187 (21.2)
Others	3211 (34.1)	12,491 (36.8)
Diagnosis of Dementia	4034 (42.8)	14,766 (43.5)
CCI^**^		
0	1149 (12.2)	3734 (11.0)
1–2	3064 (32.5)	11,763 (34.6)
3–4	1971 (20.9)	7429 (21.9)
5+	1579 (16.8)	5481 (16.1)
Unknown	1661 (17.6)	5574 (16.4)
Inpatient hospitalization at EOL^**^		
One within six months	2737 (29.0)	10,543 (31.0)
Two or more within six months	1931 (20.5)	7611 (22.4)
None within six months	1835 (19.5)	6400 (18.8)
None within two years	2921 (31.0)	9427 (27.7)
Any inpatient hospitalization within last month^**^	2291 (24.3)	9096 (26.8)

^**^ *p* < 0.01.

^a^ *Race/ethnicity* was utilized rather than race categories due to privacy concerns with cell sizes <10.

## Discussion

Families often play an important role in advocating for care at EOL; however, most existing research is limited by convenience samples with inadequate long-term follow-up data.^21^ To our knowledge, this is the first population-based study to explore the sociodemographic and death characteristics of NH decedents with and without FDF. This Utah C-PoPs study represents preliminary work deriving and describing this NH cohort and demonstrates that it is feasible to link FDF to NH decedents using a population pedigree registry.

Findings highlight gender, race, ethnicity, health insurance, socioeconomic, and geographic disparities in NH decedents without FDF—factors that may impact EOL care for an already vulnerable population.^22^ Interestingly, despite similar levels of disease burden (CCI 3+), those without FDF were less likely to have multiple hospitalizations at the EOL compared to those with FDF. Such a finding aligns with our prior mixed-methods studies, as well as other studies in the literature^7–13^ suggesting that families may advocate for more aggressive care at the EOL. While fewer hospitalizations at EOL are generally perceived as a desirable outcome, it is not clear whether this finding reflects underlying social determinants of health that impact access to care and contribute to EOL health disparities,^22^ or the significant role family may play in advocating for more aggressive care at the EOL, as other studies have found.^5,6,23–27^ Future research should examine patterns of EOL health care utilization, including NH length of stay and use of palliative and hospice care to better understand the role families play at EOL.

While 90.6% of the population in Utah lives in urban settings, ∼20% of Utah NHs are located in rural counties. Rural NHs are an important focus for policymakers given the lack of available postacute and long-term care services in these areas.^28,29^ Our study found that nearly 25% of all decedents died in a rural/frontier NH. Despite national policy initiatives to promote home- and community-based services over institutional care,^30^ these efforts have been slow to be realized for individuals living in rural areas who have limited access to NH alternatives such as formal paid care, assisted living facilities, and adult day care centers.^31,32^ Future research is needed to better understand the impact of family, or lack thereof, on rural NH resident EOL care trajectories.

We acknowledge several study limitations. First, this study utilized data from one state; therefore, findings may not generalize to the national NH population. Nevertheless, study NH population demographics mirror national estimates that NH residents are predominately female, White, with at least a high school education.^33,34^ Second, this study is our first analysis of this NH cohort, and we only examined those who died in a NH and the binary association of FDF on EOL hospitalizations. Future work will link CMS Minimum Data Set, electronic health record, administrative claims, and other data for both NH residents and their FDF to examine the effect of types of family on NH resident EOL care outcomes, and how caring for a NH resident impacts family health. Third, not all family members serve as caregivers, or they may live out of state yet still provide substantial long-distance caregiving—factors not accounted for in this study. Other studies, suggest, however, that family geographic proximity, as well as their employment status and/or own health status may impact the role they want, or can, play in the care of NH residents.^9^ Despite possible limitations, this is the first study to find that among NH decedents, those with and without FDF have different sociodemographic and death characteristics—factors that may impact care at EOL. It further sets the stage for future research understanding the critical role that family plays in EOL care experiences of NH residents.

## Conclusions

This study begins to illuminate vulnerable populations of NH residents (e.g., those without FDF and/or living in rural areas) for whom greater support may be needed to reduce health disparities and improve EOL care. These findings align with previous research suggesting NH families may need more anticipatory guidance and palliative care support to reduce potentially aggressive care at the EOL.^5,6,35,36^ Understanding the nature of FDF relationship type on NH resident EOL care trajectories and outcomes is an important next step in clarifying the role of families of persons living and dying in NHs.

## Abbreviations Used

CCI: Charlson Comorbidity Index
COPD: Chronic Obstructive Pulmonary Disease
EOL: end-of-life
FDF: first-degree family
ICD: International Classification of Diseases
IQR: interquartile range
NH: nursing home
SD: standard deviation
UPDB: Utah Population Database
Utah C-PopS: Utah Caregiving Population Science

## References

[1] Mitchell SL, Teno JM, Miller SC, et al. A national study of the location of death for older persons with dementia [published correction appears in J Am Geriatr Soc. 2005 Apr;53(4):741]. J Am Geriatr Soc 2005;53(2):299–305; doi: 10.1111/j.1532-5415.2005.53118.x 15673356

[2] National Center for Health Statistics. Health United States, 2010: With Special Feature on Death and Dying. National Center for Health Statistics: Hyattsville, MD, USA; 2011. 21634072

[3] Brock DB, Foley DJ. Demography and epidemiology of dying in the U.S. with emphasis on deaths of older persons. Hosp J 1998;13(1–2):49–60; doi: 10.1080/0742-969x.1998.11882887 9644392

[4] Bercovitz A, Decker FH, Jones A, et al. End-of-life care in nursing homes: 2004 National Nursing Home Survey. Natl Health Stat Rep 2008;8(9):1–23. 19013934

[5] Stephens C, Halifax E, Bui N, et al. Provider perspectives on the influence of family on nursing home resident transfers to the emergency department: Crises at the end of life. Curr Gerontol Geriatr Res 2015;2015:893062; doi: 10.1155/2015/893062 26379704 PMC4561315

[6] Stephens C, Halifax E, Bui N, et al. “They don't trust us”: The influence of perceptions of inadequate nursing home care on emergency room transfers and the potential role for telehealth. Clin Nurs Res 2019;21; doi: 10.1177/1054773819835015 PMC10242499 31007055

[7] Stephens CE, Hunt LJ, Bui N, et al. Palliative care eligibility, symptom burden, and quality-of-life ratings in nursing home residents. JAMA Intern Med 2018;178(1):141–142; doi: 10.1001/jamainternmed.2017.6299 29159368 PMC5833507

[8] Teno JM, Clarridge BR, Casey V, et al. Family perspectives on end-of-life care at the last place of care. JAMA 2004;291(1):88–93; doi: 10.1001/jama.291.1.88 14709580

[9] Puurveen G, Baumbusch J, Gandhi P. From family involvement to family inclusion in nursing home settings: A critical interpretive synthesis. J Fam Nurs 2018;24(1):60–85; doi: 10.1177/1074840718754314 29455580 PMC5833026

[10] Roberts AR, Ishler KJ. Family involvement in the nursing home and perceived resident quality of life. Gerontologist 2018;58(6):1033–1043; doi: 10.1093/geront/gnx108 28977636

[11] Holt-Lunstad J. Why social relationships are important for physical health: A systems approach to understanding and modifying risk and protection. Annu Rev Psychol 2018;69:437–458; doi: 10.1146/annurev-psych-122216-011902 29035688

[12] Valtorta NK, Moore DC, Barron L, et al. Older adults' social relationships and health care utilization: A systematic review. Am J Public Health 2018;108(4):e1–e10; doi: 10.2105/AJPH.2017.304256 PMC5844393 29470115

[13] Tay DL, Ornstein KA, Meeks H, et al. Evaluation of family characteristics and multiple hospitalizations at the end of life: Evidence from the Utah population database. J Palliat Med 2022;25(3):376–387; doi: 10.1089/jpm.2021.0071 34448596 PMC8968848

[14] Kelly BC, Hanson HA, Hollingshaus MS, et al. Familial and social determinants of place of death: Insights from the Utah Population Database, 1998–2016. J Am Geriatr Soc (Under review). 10.1080/07481187.2023.2255864 PMC11119959 37676820

[15] Smith KR, Fraser A, Reed DL, et al. The Utah Population Database. A model for linking medical and genealogical records for population health research. Hist Life Course Stud 2022;12:58–77; doi: 10.51964/hlcs11681

[16] Hollingshaus MS, Coon H, Crowell SE, et al. Differential vulnerability to early-life parental death: The moderating effects of family suicide history on risks for major depression and substance abuse in later life. Biodemogr Soc Biol 2016;62(1):105–125; doi: 10.1080/19485565.2016.1138395 PMC4929083 27050036

[17] Hollingshaus MS, Smith KR. Life and death in the family: Early parental death, parental remarriage, and offspring suicide risk in adulthood. Soc Sci Med 2015;131:181–189; doi: 10.1016/j.socscimed.2015.02.008 25704222 PMC4380626

[18] Smith KR, Hanson HA, Norton MC, et al. Survival of offspring who experience early parental death: Early life conditions and later-life mortality. Soc Sci Med 2014;119:180–190; doi: 10.1016/j.socscimed.2013.11.054 24530028 PMC4087105

[19] Brusselaers N, Lagergren J. The Charlson comorbidity index in registry-based research. Methods Inf Med 2017;56(5):401–406; doi: 10.3414/ME17-01-0051 29582935

[20] Charlson ME, Pompei P, Ales KL, et al. A new method of classifying prognostic comorbidity in longitudinal studies: Development and validation. J Chronic Dis 1987;40(5):373–383; doi: 10.1016/0021-9681(87)90171-8 3558716

[21] Pruchno RA, Brill JE, Shands Y, et al. Convenience samples and caregiving research: How generalizable are the findings? Gerontologist 2008;48(6):820–827; doi: 10.1093/geront/48.6.820 19139255

[22] Estrada LV, Agarwal M, Stone PW. Racial/ethnic disparities in nursing home end-of-life care: A systematic review. J Am Med Dir Assoc 2021;22(2):279.e1–290.e1; doi: 10.1016/j.jamda.2020.12.005 33428892 PMC8128037

[23] Boockvar KS, Burack OR. Organizational relationships between nursing homes and hospitals and quality of care during hospital-nursing home patient transfers. J Am Geriatr Soc 2007;55(7):1078–1084; doi: 10.1111/j.1532-5415.2007.01235.x 17608882

[24] Houttekier D, Vandervoort A, Van de Block L, et al. Hospitalizations of nursing home residents with dementia in the last month of life: Results from a nationwide survey. Palliat Med 2014;28(9):1110–1117; doi: 10.1177/0269216314535962 24866759

[25] Lamb G, Tappen R, Diaz S, et al. Avoidability of hospital transfers of nursing home residents: Perspectives of frontline staff. J Am Geriatr Soc 2011;59(9):1665–1672; doi: 10.1111/j.1532-5415.2011.03556.x 21883105

[26] Robinson CA, Bottorff JL, Lilly MB, et al. Stakeholder perspectives on transitions of nursing home residents to hospital emergency departments and back in two Canadian provinces. J Aging Stud 2012;26(4):419–427; doi: 10.1016/j.jaging.2012.06.001 22939538

[27] Simmons SF, Durkin DW, Rahman AN, et al. The value of resident choice during daily care: Do staff and families differ?. J Appl Gerontol 2014;33(6):655–671; doi: 10.1177/0733464812454010 25143465 PMC4142524

[28] RUPRI Center for Rural Health Policy Analysis. Nursing Homes in Rural America: A Chartbook. 2022. Available from: https://rupri.public-health.uiowa.edu/publications/other/Nursing%20Home%20Chartbook.pdf [Last accessed: March 13, 2023].

[29] Rural Health Information Hub. Rural Long-Term Care Facilities. 2021. Available from: https://www.ruralhealthinfo.org/topics/long-term-care [Last accessed: March 13, 2023].

[30] Kaye HS, Harrington C. Long-term services and supports in the community: Toward a research agenda. Disabil Health J 2015;8(1):3–8; doi: 10.1016/j.dhjo.2014.09.003 25445015

[31] Siconolfi D, Shih RA, Friedman EM, et al. Rural-urban disparities in access to home- and community-based services and supports: Stakeholder perspectives from 14 states. J Am Med Dir Assoc 2019;20(4):503.e1–508.e1; doi: 10.1016/j.jamda.2019.01.120 30827892 PMC6451868

[32] Tyler DA, Fennell ML. Rebalance without the balance: A research note on the availability of community-based services in areas where nursing homes have closed. Res Aging 2017;39(5):597–611; doi: 10.1177/0164027515622244 26685182 PMC4912472

[33] Tjia J, Dharmawardene M, Givens JL. Advance directives among nursing home residents with mild, moderate, and advanced dementia. J Palliat Med 2018;21(1):16–21; doi: 10.1089/jpm.2016.0473 28772095

[34] Ne'eman A, Stein M, Grabowski DC. Nursing home residents younger than age sixty-five are unique and would benefit from targeted policy making. Health Aff (Millwood) 2022;41(10):1449–1459; doi: 10.1377/hlthaff.2022.00548 36190884 PMC12927865

[35] Miller SC, Dahal R, Lima JC, et al. Palliative care consultations in nursing homes and end-of-life hospitalizations. J Pain Symptom Manage 2016;52(6):878–883; doi: 10.1016/j.jpainsymman.2016.05.017 27650008 PMC5154868

[36] Miller SC, Lima JC, Intrator O, et al. Palliative care consultations in nursing homes and reductions in acute care use and potentially burdensome end-of-life transitions. J Am Geriatr Soc 2016;64(11):2280–2287; doi: 10.1111/jgs.14469 27641157 PMC5118125

